# Bibliographical

**Published:** 1891-10

**Authors:** 


### Bibliographical.

Chemistry of the Carbon Compounds, or Organic Chemistry, by Prof. Victor von Richter, University of Breslau. Second American Edition, from the Sixth German Edition, with illustrations; authorized translation by Edgar F. Smith, Prof. of Chemistry University of Pennsylvania. Philadelphia; P. Blakiston, Son & Co., 1012 Walnut street, 1891.

The growth and expansion of chemical science is so great and so rapid, that it would seem a difficult task to make a full record of all that has developed.

Such works as the above lead us on to quite a full comprehension of what is being done in the chemical world. Perhaps no other department of science has made such rapid strides during the last half century as that of chemistry. The following words from the preface of this edition will give some idea of the scope of the volume: "The present American edition of von Richter's Organic Chemistry will be found to differ very considerably in its arrangement and size from its first edition. The introduction contains new and valuable additions upon analysis, the determination of molecular weights, recent theories on chemical structure, electrical conductivity, etc.

The section devoted to the carbonhydrates has been entirely rewritten and presents the most recent views in regard to the constitution of this interesting group of compounds. The sections relating to the trimethylene, tetramethylene and pentamethylene series, the furfurane, pyrrol and thiophene derivatives have been greatly enlarged, while the subsequent chapters devoted to the discussion of the aromatic compounds are quite exhaustive in their treatment of special and important groups."

The subject of organic chemistry is doubtless now receiving more attention than ever before, and in this volume is presented much that will stimulate the prosecution of this hitherto very difficult department of chemical science.

The volume, as arranged, will serve not only as a text book, and indeed as a reference volume, but also as a guide in conducting work in the organic laboratory. To this end numerous methods are given for the preparation of the most important and most characteristic derivatives of the different classes of bodies. The study of organic chemistry has ever been regarded as a difficult one, one fraught with, in many respects, great embarrassments. In this volume, however, many of the points are presented in so clear and understandable a manner as to be enteresting to any one having reasonable attainments in chemical science, and especially is this true in the introduction of the volume, occupying the first 67 pages. This work, while it will not, perhaps, be appreciated by the tyro in chemistry, and by those easily satisfied by what it presents, will be interesting and valuable to those who have made greater attainments in, and have a love for chemical science generally, it should be found in the library of all such.

